# Supplementary material for: Human-centred physical neuromorphics with visual brain-computer interfaces
Source: Nat Commun. 2024 Jul 29;15:6393. doi: 10.1038/s41467-024-50775-2 (PMC11289381; doi:10.1038/s41467-024-50775-2)
Supplement: Supplementary file 1 — Supplementary Information [file 41467_2024_50775_MOESM1_ESM.pdf]

Supplementary Information.
Human-Centred Physical Neuromorphics with Visual Brain-Computer Interfaces.

Gao Wang[1], Giulia Marcucci[1], Benjamin Peters[2], Maria Chiara Braidotti[1], Lars Muckli[2], Daniele Faccio[1*]
[1] *School of Physics & Astronomy, University of Glasgow, Glasgow G12 8QQ, UK*
[2] *Centre for Cognitive NeuroImaging, School of Psychology and Neuroscience, College of Medical, Veterinary and Life Sciences, University of Glasgow, 62 Hillhead Street, Glasgow, G12 8QB, UK.*
(Dated: July 17, 2024)

Supplementary Material with additional theoretical descriptions and experimental results..

Derivation of SSVEP Model Equations

We first derive Eq. (1),

$$Y_{SFG}(\omega) = \frac{\pi}{2}\tilde{\chi}^{(2)}(\omega)\left[X * X\right](\omega). \tag{1}$$

Without loss of generalization, we show here the computation of the second-order EEG signal in Eq. (2)

$$\tilde{y}(\omega) = \sum_{n=1}^{N}\tilde{\chi}^{(n)}(\omega)\mathcal{F}\left[x^n\right](\omega) = \sum_{n=1}^{N}\tilde{y}^{(n)}(\omega), \tag{2}$$

in the simplest case $M = 1$:

$$\begin{aligned}
\tilde{y}^{(2)}_{r,M=1}(\omega) &= \tilde{\chi}^{(2)}(\omega)\mathcal{F}\left[x^2_{M=1}\right](\omega) = \\
&= \tfrac{\pi}{2}\tilde{\chi}^{(2)}(\omega)\left\{2\left(A_0^2 + A_1^2\right)\delta(\omega) + \right.\\
&+ A_0^2\left[\delta(\omega - 2\omega_0) + \delta(\omega + 2\omega_0)\right] + A_1^2\left[\delta(\omega - 2\omega_1) + \delta(\omega + 2\omega_1)\right]\\
&+ \left. 2A_0A_1\left[\delta(\omega - \omega_0 - \omega_1) + \delta(\omega + \omega_0 - \omega_1) + \delta(\omega - \omega_0 + \omega_1) + \delta(\omega + \omega_0 + \omega_1)\right]\right\} \approx \\
&\approx \tfrac{\pi}{2}\left\{\tilde{\chi}^{(2)}(0)\left[2\left(A_0^2 + A_1^2\right)\delta(\omega) + 2A_0A_1\left(\delta(\omega + \omega_0 - \omega_1) + \delta(\omega - \omega_0 + \omega_1)\right)\right] + \right.\\
&+ \tilde{\chi}^{(2)}(2\omega_a)\left[A_0^2\left(\delta(\omega - 2\omega_0) + \delta(\omega + 2\omega_0)\right) + A_1^2\left(\delta(\omega - 2\omega_1) + \delta(\omega + 2\omega_1)\right) + \right.\\
&+ \left.\left. 2A_0A_1\left(\delta(\omega - \omega_0 - \omega_1) + \delta(\omega + \omega_0 + \omega_1)\right)\right]\right\}.
\end{aligned} \tag{3}$$

In Eq. (3), the function $\tilde{\chi}^{(n)}(\omega)$ is approximated to their values in $0, 2\omega_a$ because they are supposed to be symmetric with respect to $\omega = 0$ and $|\omega_{1,2} - \omega_a| \leq \mathrm{d}\omega$.
Let us now consider only $\omega > 0$. Eq. (3) reduces to

$$\begin{aligned}
\tilde{y}^{(2)}_{r,M=1}(\omega > 0) &= \tfrac{\pi}{2}\tilde{\chi}^{(2)}(\omega)\left\{A_0^2\delta(\omega - 2\omega_0) + A_2^2\delta(\omega - 2\omega_1) + 2A_0A_1\left[\delta(\omega - \omega_0 - \omega_1) + \delta(\omega + \omega_0 - \omega_1)\right]\right\} \approx \\
&\approx \tfrac{\pi}{2}\left\{\tilde{\chi}^{(2)}(0)\underbrace{\left[2A_0A_1\delta(\omega + \omega_0 - \omega_1)\right]}_{\text{DFG}} + \right.\\
&+ \left.\tilde{\chi}^{(2)}(2\omega_a)\underbrace{\left[A_0^2\delta(\omega - 2\omega_0) + A_1^2\delta(\omega - 2\omega_1) + 2A_0A_1\delta(\omega - \omega_0 - \omega_1)\right]}_{\text{SFG (including SHG)}}\right\}.
\end{aligned} \tag{4}$$

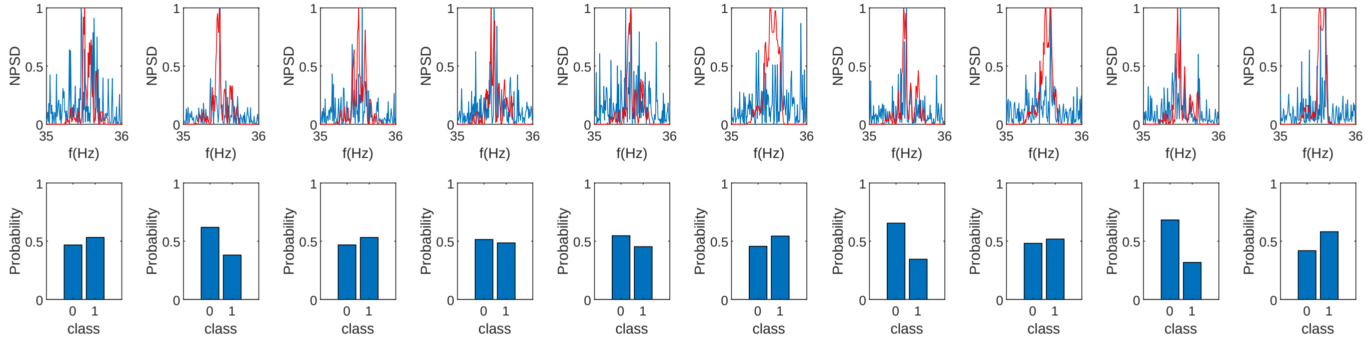

Supplementary figure 1: **BCI Image Classification: PNN Results.** Results of classification experiments of grayscale handwritten digits “0” and “1”, downsampled to 8×8 pixels. EEG-measured spectra in the SFG regime are reported in blue in the first row, while red lines represent synthetic data. Corresponding readout probability distributions over the two classes “0” and “1” are shown in the second row. Each spectrum is correctly classified.

If we consider just $\omega > \mathrm{d}\omega$, only the part referring to the SFG (with second harmonic generation, SHG, considered as an SFG from equal fundamental harmonics) remains.
The last step to derive Eq. (1) is to exploit the convolution properties of the Dirac delta functions. Indeed, from

$$X(\omega) = \sum_{m=0}^{M} A_m \delta(\omega - \omega_m), \tag{5}$$

one can see that the Dirac delta functions allows us to move easily from continuous to discrete representations and back; that is, from Eq. (1) to Eq. (4) and vice versa.
The same holds true for the case of two narrow bands:

$$\begin{aligned} Y_{SFG}(\omega) &= \tfrac{\pi}{2}\tilde{\chi}^{(2)}(\omega)\left[(X+\alpha)*(X+\alpha)\right](\omega) = \\ &= \tfrac{\pi}{2}\tilde{\chi}^{(2)}(\omega)\Bigg[\underbrace{X*X}_{\in[2\omega_a-\mathrm{d}\omega;2\omega_a+\mathrm{d}\omega]} +2 \underbrace{\alpha*X}_{\in[\omega_a+\omega_b-\mathrm{d}\omega;\omega_a+\omega_b+\mathrm{d}\omega]} + \underbrace{\alpha*\alpha}_{\in[2\omega_b-\mathrm{d}\omega;2\omega_b+\mathrm{d}\omega]}\Bigg](\omega), \end{aligned} \tag{6}$$

which implies

$$Y_{X+\alpha}(\omega) = \frac{\pi}{2}\tilde{\chi}^{(2)}(\omega)\left[X*\alpha\right](\omega), \tag{7}$$

Single-Layer SSVEP BCI Classifier: Further Results

The results of the handwritten digit image classification using our SSVEP-based BCI are more broadly presented in Fig. 1, demonstrating the effectiveness and limits of our approach in classifying handwritten digits “0” and “1”.
As further proof of the computational capabilities of our SSVEP-based BCI, we demonstrate its performance as a classifier of a breast cancer multivariate dataset [1], to underline the versatility of our PNN in dealing with different data,

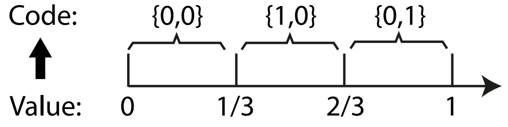

Supplementary figure 2: **Breast Cancer Dataset Encoding.** Strategy to encode the biopsy normalized parameters - such as the feature vector shown in Table I - in the first three classes of binary numbers.

No	Name	Value	Code
0	Bias	0.50	{1,0}
1	Clump thickness	0.44	{1,0}
2	Uniformity of cell size	1.00	{0,1}
3	Uniformity of cell shape	1.00	{0,1}
4	Marginal Adhesion	0.22	{0,0}
5	Single epithelial cell size	0.67	{1,0}
6	Bare nuclei	0.22	{0,0}
7	Bland chomatin	0.78	{0,1}
8	Normal nucleoli	1.00	{0,1}
9	Mitoses	0.11	{0,0}

Table I: Example of one breast cancer dataset feature vector, extracted from a biopsy sample, and its encoding into a binary vector, as further detailed in Fig. 2. The first term, i.e. bias, is arbitrarily added.

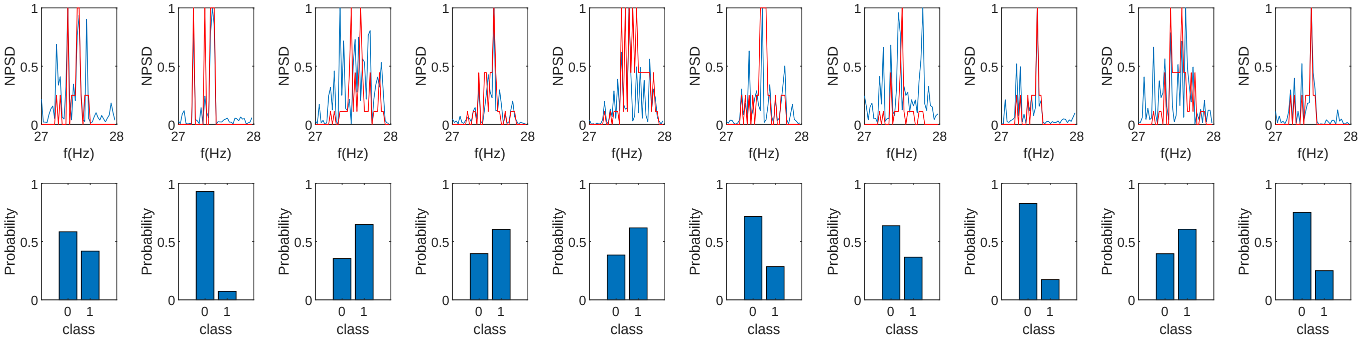

Supplementary figure 3: **BCI Breast Cancer Multivariate Dataset Classification: PNN Results.** Results of classification experiments of *benign* and *malignant* cancers. EEG-measured spectra in the SFG regime are reported in blue in the first row, while red lines represent synthetic data. Corresponding readout probability distributions over the two classes *benign* (labelled "0") and *malignant* (labelled "1") are shown in the second row. Only the seventh spectrum is not correctly classified.

from images to multivariate numerical lists. Each datum in the breast cancer dataset is a numerical 9-dimensional feature vector extracted from a biopsy sample, and labelled either as *benign* or as *malignant*, depending on the nature of the analyzed breast cancer.

To convert these feature vectors into an frequency encoded signal, a series of preprocessing steps are undertaken, as partially illustrated in Table I and Fig. 2. The initial step involves resizing each feature, denoted as x, to a rescaled range of $[0, 1]$ as $\frac{x-x_{min}}{x_{max}-x_{min}}$, such that white represents the NPSD normalization constant, black denotes the average background noise, and intermediate gray tones are determined proportionally. Specifically, here x_{min} and x_{max} represent the minimum and maximum values within the entire dataset range. Subsequently, a vector quantization process is applied, employing a 2-bit codebook composed of {00, 10, 01} and a partition scheme of $\{0, \frac{1}{3}, \frac{2}{3}, 1\}$ as depicted in Fig. 2. Additionally, a bias feature, coded as {10}, is appended to the data to ensure representation in the event that all feature vectors are encoded as zeros. For instance, if the original feature values are {0.1, 0.4, 0.9}, the resulting transformed feature values will manifest as a binary vector {10001001}. Ultimately, this conversion process transforms the original 9×1 feature vectors into binary feature vectors with dimensions of 20×1. During testing, the encoding extends to both the testing data and control parameters, where they are encoded as narrowband stimuli utilized in LED modulation at frequencies of approximately 12 Hz and 15 Hz, respectively. The outcomes of these encoding processes are visualized in Fig. 3.

As an additional application of our BCI setup, we show that even a feedforward RC architecture, that is, an Extreme learning machine (ELM) [2], can be realized. In this context, we revisit the multivariate breast cancer dataset classification task. However, in this iteration, we eliminate the dependency on genetically algorithm-optimized control parameters, opting instead for encoding based solely on input data information. Decoding is accomplished by evaluating the squared absolute value of the output NPSD within the SHG regime. Results are presented in Fig. 4.

Figure 5 shows the statistical distribution of the classification results in the two layer PNN task described in the main article, where a total of 13 participants have been tested as the second layer of the 2-layer PNN classifier. These

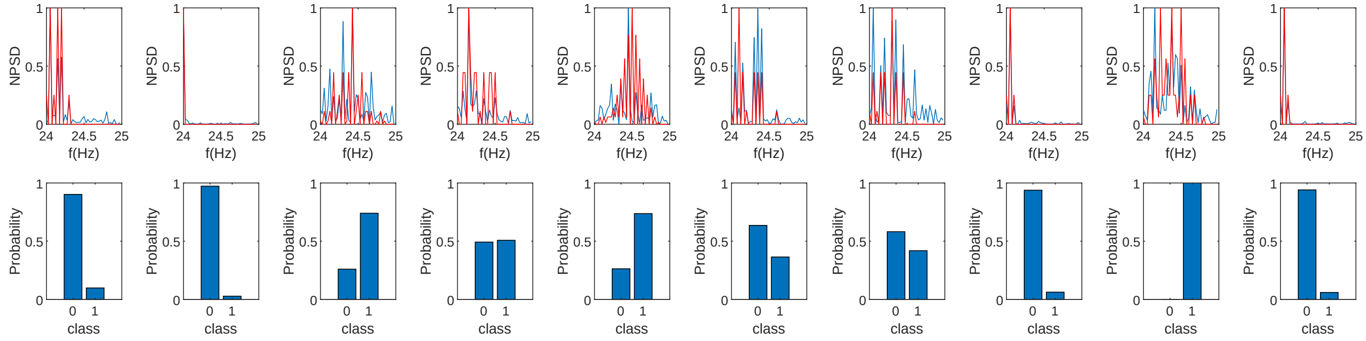

Supplementary figure 4: **BCI Breast Cancer Multivariate Dataset Classification: ELM Results.** Results of classification experiments of *benign* and *malignant* cancers. EEG-measured spectra in the SHG regime are reported in blue in the first row, while red lines represent synthetic data. Corresponding readout probability distributions over the two classes *benign* (labelled "0") and *malignant* (labelled "1") are shown in the second row. Only the seventh spectrum is not correctly classified.

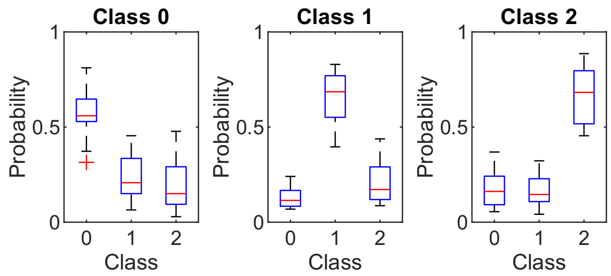

Supplementary figure 5: **Multi-layer PNN Probability box plot.** 13 subjects were tested in total and the Probability distribution for each different class is illustrated by the box plot. After considering all samples, the t-test between "Target class" and "Others class" shows $t(13) = 9.77, p = 6 \times 10^{-12}$.

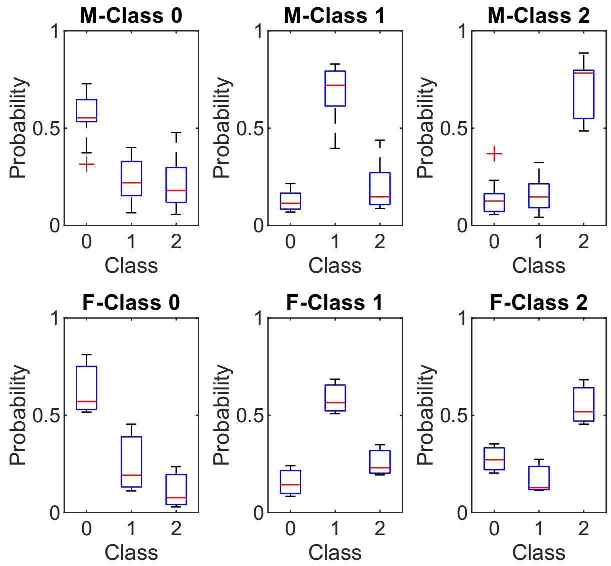

Supplementary figure 6: **Multi-layer PNN Probability box plot, disaggregated by sex.** Top row refers to male only (10 subjects) and bottom row to female only (3 subjects). No significant difference in performance is observed in terms of the final PNN classification fidelity, i.e. all three classes are correctly classified in all cases.

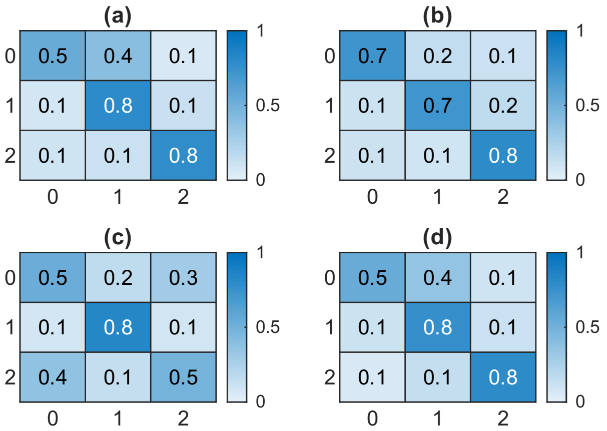

Supplementary figure 7: **Multi-layer PNN Probability Tables.** Classification probability distributions for different combinations of multi-layer PNN experiments on four participants for the same image classification task, i.e., classification of 3 classes Iris flower, “0”, “1”, and “2”. In these tables, the y-axis represents the target value to be predicted, and the x-axis represents the classes of the readout probability distributions for testing experiments whose first layer is Participant number 1 and the second layer is (a) Participant number 1, (b) Participant number 2, (c) Participant number 3, (d) Participant number 4. Network training is performed employing synthetic data produced by the same phenomenological model described in Methods.

results show that the ability to use the visual system as a classifier, also in a multi-layer PNN, is generic and is not limited to specific individuals.

Figure 6 shows the same results as Figure 5, now disaggregated by sex. Both male and female participants correctly classify all classes with similar fidelity.

Finally, Figure 7 shows an example of the classification probabilities for the two-layer PNN with the same participant (a) or with participant 1 for the first layer and 3 other participants for layer 2 (b-d). These illustrate similar behaviour in all cases, indicating the possibility to generalise this approach to different people.

Information Transmission Rates.

We can evaluate the total information transmitted by the BCI system in various ways. For example, a very simple approach, presented in the main text, simply assumes that the final image is perfectly reconstructed, i.e. matches the original input image. We also assume in all of the following that each pixel is an independent channel (this therefore reflects the experimental choice that each pixel is encoded in one single frequency term and that this information does not spread over adjacent channels). We also assume that each pixel has a binary distribution, black or white, i.e. each pixel encodes 1 bit of information.

We now refer explicitly to the data in Fig. 2 in the main text in order to estimate the information transmitted for a specific case. Each image has 196 pixels, therefore 196 bits. If we specifically focus on Fig. 2(p), we first assume that the (binarized) reconstructed image matches the original image exactly, then 196 bits were transmitted. As the image was transmitted in 16.3 seconds, the information transfer rate (ITR) is 196 bits/ 16.3 seconds = 12 bits/second. This is the result reported in the main paper.

The transmission, of course, introduced reconstruction errors. Therefore, the rate of 12 bits/seconds, which assumes no transmission error, is an overestimate of the actual bit rate. A more precise evaluation would require us to also consider the possibility for some pixels to actually not have the correct value after binarisation. This can be accounted for by calculating the Mutual Information (MI) between the original image X and the reconstructed (binarized) image Y, which is defined as $I(X;Y) = H(X) - H(X|Y)$. Here, $H(X)$ is the marginal entropy (i.e. entropy of the input image) and $H(X|Y)$ is the conditional entropy (i.e. the uncertainty about the original image X, that is left after knowing the reconstructed image Y). We determine the MI for one pixel (i.e., $H(X) = 1$ bit) and estimate $H(X|Y)$ from the normalized error matrix between true pixel values and reconstructed (binarized) pixel values across the full image (i.e., $P(X,Y)$). The total MI is then 196 times the MI for one pixel.

Figure 8(a) reports the mutual information based on our measurements for increasing integration time, showing a general increase of the total mutual information as the integration times become longer. This is in keeping with

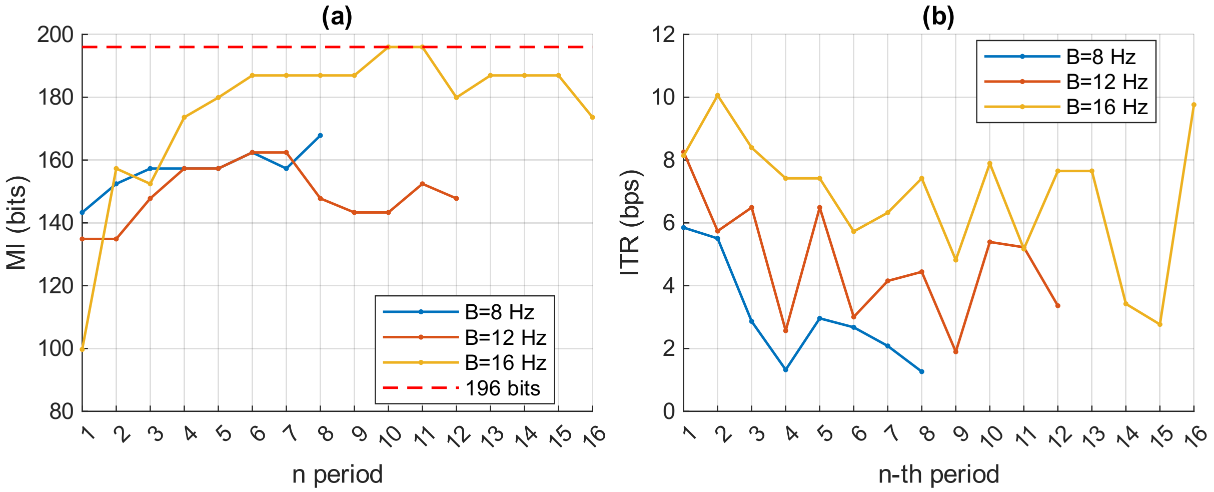

Supplementary figure 8: **Information analysis.** (a) Mutual information for various images shown in Figure 2 (main paper). The maximum possible information is 196 bits (corresponding to the input image information). The mutual information (MI) also accounts for the pixels that are not correctly reconstructed. 'B' indicates the total bandwidth used for the SSVEP encoding and 'n period' indicates the number of transmission periods T, i.e. sequential repetitions of the full image transmission. The MI generally increases with the number of periods due to better SNR with longer acquisition times. (b) Information transmission rate (bits/second) for each single measurement n^{th} period T within a multi-period acquisition. We find an maximum ITR of 10 bits/second.

the results in Figure 2 of the main paper where single-period measurements are seen to lead to less precise image reconstructions compared to longer, multi-period measurements (compare Figure 2(p) with Figure 2(m)).
Figure 8(b) shows the 'time-resolved' information transfer rate (ITR) for each nth period T within a longer measurement on many periods (up a maximum acquisition of 196 seconds). The ITR for Figure2(p) considered above, is now 8.3 bits/second - this value is lower compared to the approximate calculation given above as a result of MI also accounting for pixels that are incorrectly reconstructed. Overall, Figure 8(b) shows that across all of our measurements, we have maximum ITR of 10 bits/second. We also note a slight downward trend in the ITR over time (the nth period occurs at time=$n * T$). This is possibly related the participant becoming tired or losing focus over longer times.

* Correspondence email address:
daniele.faccio@glasgow.ac.uk

[1] Kelly, M., Longjohn, R. & Nottingham, K. The UCI machine learning repository. URL `https://archive.ics.uci.edu`.

[2] Huang, G. B., Zhu, Q. Y. & Siew, C. K. Extreme learning machine: Theory and applications. *Neurocomputing* **70**, 489–501 (2006). URL `https://doi.org/10.1016/j.neucom.2005.12.126`.
